# Tumour location within the breast: Does tumour site have prognostic ability?

**DOI:** 10.3332/ecancer.2015.552

**Published:** 2015-07-13

**Authors:** Seth Rummel, Matthew T Hueman, Nick Costantino, Craig D Shriver, Rachel E Ellsworth

**Affiliations:** 1 Clinical Breast Care Project, Windber Research Institute, Windber, Pennsylvania 15963, USA; 2 Clinical Breast Care Project, Murtha Cancer Centre, Walter Reed National Military Medical Centre, Bethesda, Maryland 20889, USA

**Keywords:** breast quadrants, tumour location, prognosis

## Abstract

**Introduction:**

Tumour location within the breast varies with the highest frequency in the upper outer quadrant (UOQ) and lowest frequency in the lower inner quadrant (LIQ). Whether tumour location is prognostic is unclear. To determine whether tumour location is prognostic, associations between tumour site and clinicopathological characteristics were evaluated.

**Materials and Methods:**

All patients enrolled in the Clinical Breast Care Project whose tumour site—UOQ, upper inner quadrant (UIQ), central, LIQ, lower outer quadrant (LOQ)—was determined by a single, dedicated breast pathologist were included in this study. Patients with multicentric disease (*n* = 122) or tumours spanning multiple quadrants (*n* = 381) were excluded from further analysis. Clinicopathological characteristics were analysed using chi-square tests for univariate analysis with multivariate analysis performed using principal components analysis (PCA) and multiple logistic regression. Significance was defined as *P* < 0.05.

**Results:**

Of the 980 patients with defined tumour location, 30 had bilateral disease. Tumour location in the UOQ (51.5%) was significantly higher than in the UIQ (15.6%), LOQ (14.2%), central (10.6%), or LIQ (8.1%). Tumours in the central quadrant were significantly more likely to have higher tumour stage (*P* = 0.003) and size (*P* < 0.001), metastatic lymph nodes (*P* < 0.001), and mortality (*P* = 0.011). After multivariate analysis, only tumour size and lymph node status remained significantly associated with survival.

**Conclusions:**

Evaluation of tumour location as a prognostic factor revealed that although tumours in the central region are associated with less favourable outcome, these associations are not independent of location but rather driven by larger tumour size. Tumours in the central region are more difficult to detect mammographically, resulting in larger tumour size at diagnosis and thus less favourable prognosis. Together, these data demonstrate that tumour location is not an independent prognostic factor.

## Background

Although mortality rates from breast cancer have decreased since 1990, it is estimated that >39,500 women in the United States died from breast cancer in 2013 [1]. A number of pathological characteristics have been identified, which can be used as prognostic factors, including tumour size and grade, hormone receptor, HER2, and lymph node status [2, 3], which are all considered when predicting prognosis and determining the most effective treatment options. In addition, molecular tests have been developed that add prognostic value by determining intrinsic subtype, stratification of tumours into low or high grade, or predicting recurrence [4–7]. Despite these factors and tests, predicting patient outcome is imprecise, as patients with similar pathological characteristics treated with identical regimens have highly variable clinical outcomes [8], suggesting that identification of additional prognostic factors may improve patient stratification.

Tumour location within the breast has been proposed as an independent prognostic factor. For example, the frequency of axillary lymph node metastasis was significantly lower in the upper inner quadrant (UIQ, 20.6%) compared to all other quadrants (33.2%) [9]. In contrast, tumours in the upper outer quadrant (UOQ), which is the most frequent site of tumour location, have been associated with improved survival compared to other quadrants [10, 11]; survival data for non-UOQ tumours have been mixed with findings demonstrating decreased survival for the lower inner quadrant (LIQ), and lower, medial or periareolar regions [12–17]. In contrast, other studies have found no association between tumour location and outcome [18–20].

Reasons for discordant results are varied. For example, the manner in which tumour locations are grouped can affect survival results: when quadrants were evaluated individually, patients with tumours in the LIQ had a twofold increase in mortality; however, using the same data set, when tumour sites were combined into inner versus outer or lower versus upper, survival did not differ [13]. Other studies have suggested that the less favourable outcomes associated with tumours in the medial region can be attributed to undetected positive lymph nodes in internal mammary lymph nodes [12, 13], although several studies have demonstrated that treatment of the internal mammary lymph nodes does not improve survival [20–23]. Finally, tumour locations demonstrating less favourable survival are associated with known prognostic factors such as increased tumour size, grade, or lymph node status [13, 16]. Thus, to determine whether tumour location is an independent prognostic factor, data from the Clinical Breast Care Project (CBCP) was analysed to determine whether tumour site was associated with any clinicopathological characteristics.

## Methods

### Patient enrolment and consent

For inclusion in the CBCP, all patients met the following criteria: (1) adult over the age of 18 years, (2) mentally competent and willing to provide informed consent, and (3) presenting to the breast centres with evidence of possible breast disease. Tissues were collected with approval from the Walter Reed National Military Medical Centre (WRNMMC) Human Use Committee and Institutional Review Board. All enrolled subjects voluntarily agreed to participate and were provided with layered consent forms that included permission to gather tissue and that described the primary research uses of the samples.

### Pathological characterisation

The CBCP database was queried to identify all patients with known tumour location diagnosed 2001–2013. Tumour location was classified using the SEER Coding guidelines (http://seer.cancer.gov/archive/manuals/2010/AppendixC/breast/coding_guidelines.pdf). Pathological characterisation of all specimens was performed by a single, dedicated breast pathologist as previously described [24]. To ensure consistency, diagnosis of all tumour samples were made by one pathologist from haematoxylin and eosin stained slides; staging was performed using guidelines defined by the AJCC *Cancer Staging Manual* seventh edition [25] and grade was assigned using the Bloom–Richardson system of classification [26, 27]. Oestrogen receptor (ER), progesterone receptor (PR), and HER2 status were determined by immunohistochemical (IHC) analysis (MDR Global, Windber, PA); the cut-off for defining ER and PR positivity was ≥1% positively staining cells following the ASCO/CAP guildeines [28] while cases with HER2 scores = 2+ were then evaluated by fluorescence *in situ* hybridisation using the PathVysion® HER-2 DNA Probe kit (Abbott Laboratories, Abbott Park, IL).

### Statistical Analysis

Univariate analysis was performed using chi-square analysis (http://www.physics.csbsju.edu/stats/contingency_NROW_NCOLUMN_form.html). After initial univariate analysis revealed highly associated variables, a multivariate approach was used to determine the extent to which each could be considered an independent prognostic factor. In the initial multivariate analysis, seven possible predictor variables were included: lymph node status, PR status, ER status, HER2 status, location, tumour grade, and size. Principal components analysis (PCA) was performed in order to reduce the dimensionality of a subsequent regression model and to create uncorrelated variables. Multiple logistic regression analysis was performed to assess the influence of these components on patient survival. The informative components were then decomposed to determine the true independent predictors of patient survival resulting from the regression. Further multiple logistic regression analysis was performed on lymph node status, tumour size, and tumour grade in order to determine how strongly other characteristics were associated with these variables.

Because our variables were categorical, one category for each was established as the reference point to which the other categories were compared in regression modelling. The reference categories corresponded to a negative status within the lymph node, PR, ER, and HER2 variables, the central region for tumour location, tumour grade 1, and tumour size T1. The odds ratios established through regression for the complementary categories represent the relative odds that a change from the reference category to the alternative category would cause a change in the dependent variable when all other variables are held constant. Statistical significance was defined as *P* < 0.05. Statistical analysis was carried out using R version 3.1.1 (http://www.R-project.org/).

## Results

### Patient characteristics

**Figure 1.**
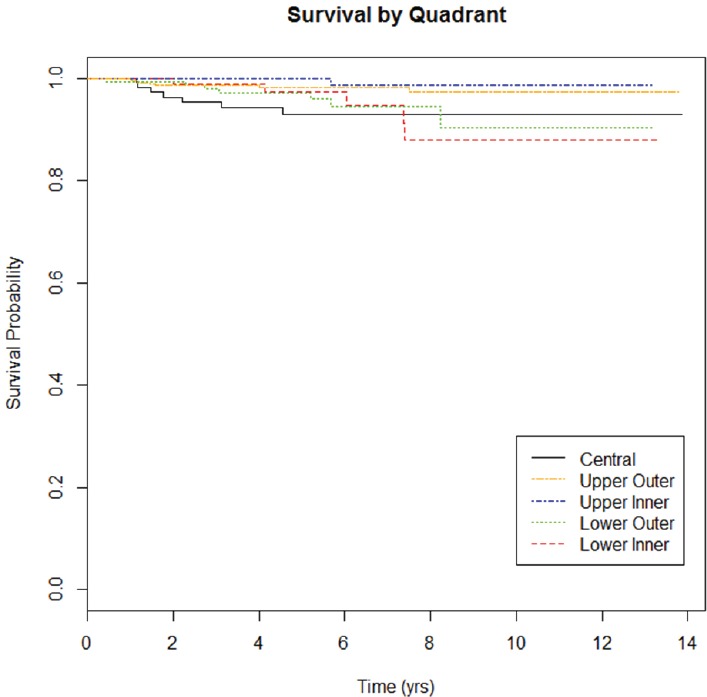
Survival curve by tumour location. Median time to death was 1.52 years for UOQ, 5.69 years for UIQ, 3.08 years for LOQ, 6.03 years for LIQ, and 1.77 years for central.

As of July 2014, 1,483 patients were enrolled in the CBCP. Of these, 122 were diagnosed with multicentric disease, and 381 had tumour locations spanning multiple quadrants (e.g., mid-inner), leaving 980 eligible patients for this study. The average age at diagnosis was 58.7. The majority of patients were Caucasian (80%), and the majority of tumours were early stage (88%), ER+HER2− (72%), T1 (66%) with negative lymph nodes (70%). Tumour grade was split into well-differentiated (29%), moderately differentiated (38%) and poorly differentiated (33%). Twenty-nine patients have died of disease (Figure 1). Median follow-up by tumour location was 5.3 years (range 1.6–13.8) for UOQ, 5.4 years (range 1.2–13.2) for UIQ, 5.6 years (range 2.0–13.2) for LOQ, 5.8 years (range 1.9–13.3) for LIQ, and 5.6 years (range 1.7–13.9) for central.

### Differences in pathological characteristics by tumour location

Tumour location was higher in the UOQ (51.5%) compared to the UIQ (15.6%), lower outer quadrant (LOQ, 14.2%), central (10.6%), or LIQ (8.1%). To evaluate whether breast cancer risk factors are associated with tumour location, age at diagnosis, ethnicity, body mass index (BMI), and breastfeeding were evaluated by quadrant (Table 1); none of these factors differed significantly. Analysis of pathological factors revealed significant differences for tumour stage, size, lymph node status, and survival (Table 2), with central quadrants having a higher frequency of late-stage tumours (25%) compared to the other quadrants (range 7–13%), T3 tumours (16%) compared to other quadrants (range 0–5%) and metastatic lymph nodes (51%) compared to other quadrants (range 24–38%). Breast cancer mortality rates were highest in patients with tumours in the central quadrant (7%) compared to other quadrants (range 1–5%).

Given the linear relationship between tumour size and lymph node involvement [29], lymph node status was compared within tumour size groups T1, T2, or T3. The frequency of positive lymph nodes did not differ significantly by tumour location for any of the sizes. Multivariate analysis was performed using all of the pathological characteristics; only lymph node status and tumour size remained significant predictors of breast cancer mortality with lymph node metastases and T3 tumour size having expected odds ratios of 3.5 (*P* = 0.026) and 5.1 (*P* = 0.019), respectively. Tumour size was significantly associated with positive lymph node status with T2 and T3 tumours having expected odds ratios of 12.3 (*P* < 0.001) and 24.7 (*P* < 0.001); high-grade tumours were also associated with positive lymph node status with an expected odds ratio of 1.6 (*P* = 0.045). Tumour location was only associated with tumour size, with all quadrants having smaller tumour size compared to the central region (expected odds ratios range (0.30–0.41).

## Discussion

Determination of whether tumour location can be used prognostically is important in optimising treatment. Tumour location is highest in the UOQ (50–58%) across multiple populations, including Chinese, Danish, the United Kingdom and women treated within the United States Department of Defence healthcare system [10, 11, 14, 30]. Two studies suggest that the frequency of tumours in the UOQ has increased over time [30, 31]; given the association of tumour in the UOQ with improved prognosis, these data would suggest a trend towards a reduction in breast cancer mortality. Tumour occurrence in the LIQ, however, has also increased significantly and tumour location in the LIQ has been associated with >2-fold increase in mortality [13], suggesting that the decrease in mortality associated with increasing tumour location in the UOQ may be offset by concomitant increases in the LIQ. In our study, tumours in the UOQ showed a trend towards favourable prognosis although this did not reach the level of significance (*P* = 0.0754).

The less favourable prognosis seen in patients with tumours in the central region can be attributed to increased tumour size and positive lymph node status. Tumour size is thought to reflect the chronological age of the tumour, with smaller tumours being resected earlier than larger tumours [32]. Tumour size has been associated with positive lymph node status in multiple studies [29, 33–41]. In addition, tumour size is also prognostic in patients with both negative and positive lymph node status [42–46]. Thus, tumour location in the central region is a surrogate for larger tumour size, resulting in increased rates of metastatic lymph node and breast cancer mortality.

Previous studies have demonstrated that tumours within the central region are harder to detect than at other sites [47, 48] and that tumours in this region are more easily detected by clinical examination than mammography [49]. This difficulty in detecting tumours in the central region have been attributed to overpenetration of X-rays in the nipple-areolar complex; accurate diagnosis in this region may require the use of multiple imaging modalities [50]. The difficulty in mammographic detection and more frequent occurrence of palpable tumours in the central region explains the larger tumour size, and hence increased mortality found in our study.

Although this study included data from 980 patients, two other studies evaluated much larger data sets including tumour registry data from the US Department of Defence (*n* = 13,984) or Danish Breast Cancer Cooperative Group (*n* = 35,319). In both studies, UOQ was associated with more favourable prognosis, with one study reporting a hazard ratio of 0.820 and the other a 20% improved survival in patients whose tumours were in the UOQ [10, 11]. Our data demonstrated a trend towards improved prognosis for patients with tumours in the UOQ (expected odds ratio = 0.2988, *P* = 0.0754) when compared to the central region; this survival advantage may have reached the level of significance with a larger sample size. In addition, the median follow-up for these patients was 5.5 years; thus, differences in late recurrence (>5 years after diagnosis) by tumour location may not have been detected. Finally, treatment data for each patient were not available. While some studies suggest that less favourable prognosis by tumour location has been associated with under-staging and suboptimal treatment of tumours in the LIQ when lymph node status of the internal mammary chain is not evaluated [12, 15], others studies found that treatment differences were not responsible for the decreased survival for patients with tumours in the LIQ [13, 21, 22]. Without these data in our study, it is not possible to determine whether the less favourable prognosis for patients with tumours in the central region, or conversely, the improved prognosis for those with tumours in the UOQ, reflects differential provision of or response to treatment.

This study does have several advantages. Tumour location was specified by a single, dedicated breast pathologist, which provides a level of consistency not available in tumour registries or databases with pathological data compiled from multiple pathologists. In addition, our data were analysed by quadrant site, rather than grouped into lateral/medial [14, 15], which studies have shown may dilute the ability to detect associations between pathology and tumour location [13]. Finally, while we included tumours from the central region in this study, other studies did not include this region in statistical analysis [11, 15].

## Conclusions

In this study, the UOQ, which was the most common site for tumours within the human breast, was not significantly associated with improved survival. In addition, although central tumour location was associated with higher mortality rates on univariate analysis, multivariate analysis demonstrated that centrality was not an independent risk for survival but rather, the association with less favourable prognosis was attributable to a higher frequency of T3 tumours. Difficultly in imaging and detecting tumours within the central region leads to delayed detection and larger tumour size at diagnosis, and tumour size ≥5 cm (T3) is an independent prognostic factor for both lymph node metastases and poor outcome. Together, these data suggest that tumour location is not prognostic.

## Conflicts of interest

The author(s) declare that they have no conflict of interest.

## Authors’ contributions

Seth Rummel collected the data and performed preliminary analyses; Nick Constantino performed multivariate analysis, Matthew T Hueman and Craig D Shriver provided clinical input and reviewed the manuscript, Rachel E Ellsworth conceptualised the study and drafted the manuscript.

## Figures and Tables

**Table 1.** Comparison of risk factors at diagnosis between tumour locations. Data are presented as proportion of individuals within each category.

	UOQ (*n* = 520)	UIQ (*n* = 158)	LOQ (*n* = 143)	LIQ (*n* = 82)	Central (*n* = 107)	*P* value
**Age at Diagnosis**						
<40 years	0.06	0.04	0.05	0.07	0.03	0.115
40–49 years	0.20	0.22	0.29	0.12	0.20	
≥50 years	0.74	0.74	0.66	0.81	0.77	
**Menopausal status**						
Pre-menopausal	0.29	0.28	0.36	0.25	0.23	0.190
Post-menopausal	0.71	0.72	0.64	0.75	0.77	
**Ethnicity**						
African American	0.20	0.21	0.17	0.21	0.13	0.420
Asian	0.01	0.03	0.01	0.01	0.03	
Hispanic	0.01	0.01	0.01	0.01	0.04	
Other	0.01	0.01	0.01	0.00	0.00	
Caucasian	0.77	0.74	0.80	0.77	0.80	
**BMI**						
<18.5	0.01	0.01	0.01	0.01	0.01	0.247
18.5–24.9	0.32	0.24	0.31	0.25	0.38	
25–29.9	0.29	0.36	0.27	0.34	0.37	
≥30	0.38	0.39	0.41	0.40	0.24	
**Breastfeed**^a^						
Yes	0.46	0.44	0.47	0.53	0.49	0.616
No	0.54	0.56	0.53	0.47	0.51	

a Breastfeeding was considered only in parous women.

**Table 2.** Pathological characteristics at diagnosis by tumour location. Data are presented as proportion of individuals within each category.

	UOQ (*n* = 520)	UIQ (*n* = 158)	LOQ (*n* = 143)	LIQ (*n* = 82)	Central (*n* = 107)	*P* value
**Stage**						
I	0.56	0.66	0.53	0.59	0.46	**0.003**
II	0.33	0.27	0.34	0.30	0.29	
III	0.09	0.06	0.11	0.06	0.18	
IV	0.02	0.01	0.02	0.05	0.07	
**ER/HER2 status**						
ER+/HER2−	0.70	0.76	0.68	0.76	0.71	0.124
ER+/HER2−	0.15	0.08	0.10	0.05	0.07	
ER−/HER2+	0.05	0.10	0.07	0.04	0.06	
ER−HER2−	0.10	0.06	0.15	0.15	0.16	
**PR Status**						
Positive	0.62	0.71	0.67	0.63	0.64	0.248
Negative	0.38	0.29	0.33	0.37	0.36	
**Lymph node status**						
Negative	0.64	0.76	0.62	0.66	0.49	**<0.001**
Positive	0.36	0.24	0.38	0.34	0.51	
**Tumour Grade**						
Low	0.29	0.33	0.27	0.26	0.27	0.668
Intermediate	0.37	0.33	0.41	0.43	0.45	
High	0.34	0.34	0.32	0.31	0.28	
**Ki67**						
<20%	0.58	0.55	0.50	0.42	0.50	0.174
≥20%	0.42	0.45	0.50	0.58	0.50	
**Tumour Size**						
T1	0.67	0.71	0.70	0.71	0.52	**<0.001**
T2	0.28	0.25	0.27	0.29	0.32	
T3	0.05	0.04	0.03	0.00	0.16	
**Angiolymphatic invasion**						
Absent	0.77	0.78	0.73	0.79	0.68	0.272
Present	0.23	0.22	0.27	0.21	0.32	
**Dead of Disease**						
Yes	0.02	0.01	0.05	0.05	0.07	**0.011**
No	0.98	0.99	0.95	0.95	0.93	
